# Dataset on water quality monitoring from a wireless sensor network in a river in Kosovo

**DOI:** 10.1016/j.dib.2022.108486

**Published:** 2022-07-25

**Authors:** Figene Ahmedi, Lule Ahmedi

**Affiliations:** a Hydrotechnics and Environmental Engineering, University of Prishtina, Republic of Kosovo; b Computer Engineering, University of Prishtina, Republic of Kosovo

**Keywords:** Water quality monitoring, Surface water, Wireless sensor networks, Data engineering and data analysis

## Abstract

This dataset was collected as part of the InWaterSense project with a wireless sensor network (WSN) installed in a site in river Sitnica in Kosovo, as a case study for monitoring remotely, continuously and in real-time the surface water quality in Kosovo and how it can be extended to all surface waters in the country for quality assurance. Values of four water quality parameters are provided in the dataset, i.e., temperature, electrical conductivity, pH, and dissolved oxygen measured by respective static sensors of WSN in the time frame between May 2015 to beginning of January 2016 and every 10 min, counting to slightly over 100k measurement records in total. The dataset is hosted at the Mendeley Data repository (Ahmedi and Ahmedi 2021), and is related to the research article entitled “InWaterSense: An Intelligent Wireless Sensor Network for Monitoring Surface Water Quality to a River in Kosovo” (Ahmedi et al., 2018). The reuse potential of the dataset to the scientific community is widespread, from environmental engineering to artificial intelligence to the health sector just to mention few. Moreover, practitioners might benefit from this dataset in driving forth the pollution prevention policies and techniques.

Data were acquired measuring water quality using static sensors installed as part of a wireless sensor network in Sitnica river in the Plemetin village near Prishtina, then transmitted to the gateway device also in Plemetin via the ZigBee protocol, and finally transmitted remotely via GPRS to the server machine in the premises of the University of Prishtina.

The data received from sensors are in real-time stored in the MS SQL server.

**Specifications Table**

**Table:** 

Subject	Environmental Engineering
Specific subject area	Water Quality Monitoring
Type of data	Tables in csv and Excel files. Code in SQL script
How data were acquired	Data were acquired measuring water quality using static sensors installed as part of a wireless sensor network in Sitnica river in the Plemetin village near Prishtina, then transmitted to the gateway device also in Plemetin via the ZigBee protocol, and finally transmitted remotely via GPRS to the server machine in the premises of the University of Prishtina. The data received from sensors are in real-time stored in the MS SQL server. The WSN technology used is commercial by Libelium vendor and based on the Waspmote Plug and Sense model, an open source wireless sensor network platform: http://www.libelium.com/development/plug-sense/documentation/waspmote-plug-sense-quick-overview/
Data format	Raw and filtered data from sensors
Description of data collection	The river water quality parameters measured remotely by the WSN static sensors are: temperature, electrical conductivity, pH, and dissolved oxygen.
Data source location	SItnica river bank, Plemetin village, Municipality of Obiliq, close to capital city Prishtina, Republic of Kosova. Measurements were conducted in two points in the river: sensing node 1 (housing) and sensing node 2 (manhole) in a distance of around 100 m from each other. The GPS coordinates (longitude, latitude) of each of the two sensing nodes are: Node 1 coordinates (21.03843117, 42.70670319), Node 2 coordinates (21.03802872, 42.70727921).
Data accessibility	Repository name: Mendeley Data. Data identification number: 10.17632/krzv3g6d5f.1 Direct URL to data: https://data.mendeley.com/datasets/krzv3g6d5f/1.F. Ahmedi, L. Ahmedi, InWaterSense Dataset: Data from a wireless sensor network on water quality monitoring in a river in Kosovo, Mendeley Data, v1, 2021. http://dx.doi.org/10.17632/krzv3g6d5f.1 [dataset] [1]
Related research article	F. Ahmedi, L. Ahmedi, B. O'Flynn, A. Kurti, S. Tahirsylaj, E. Bytyçi, B. Sejdiu, A. Salihu, InWaterSense: An Intelligent Wireless Sensor Network for Monitoring Surface Water Quality to a River in Kosovo, Int. J. of Agric. and Environ. Inf. Syst. 9(1) (2018) 39-61. 10.4018/978-1-5225-5978-8.ch003. [2]

**Value of the Data**

• The usefulness of this dataset to the scientific community is manifold, namely: (1) it reflects a sample dataset gathered by a WSN remote, continuous and real-time monitoring of a surface water quality in a developing country like is the case study presented in this paper of a river in Kosovo; (2) it is even at a global scale a rare available dataset gathered by applying remote wireless sensor network in a river for monitoring its water quality with such time comprehension in frequency and continuity.
• The scientific community in the fields of environmental engineering, more specifically water quality monitoring, water resources management, then wireless sensor networks, data engineering and data analysis can benefit from these data by using them in various research tasks such as water quality trends analysis and prediction, anomalies detection in water quality data, or relatedness of water quality data to other environmental data as well as to health data just to mention few among potential multidisciplinary research tasks upon these data.
• The dataset can be enriched with semantics annotated by environmental/water experts to conduct various experiments in the field of intelligent water quality monitoring and water resources management, or in general in artificial intelligence and Internet of Things research and innovative solutions in practice for the good of society.
• Of a practical relevance, environmental agencies, decision makers and other stakeholders can in particular use these data to prevent pollution of surface waters by building decision models based on water quality classification and pollution sources detection upon these and supplementary data.

It is important to note that this dataset represents experimental raw data of water quality parameters as measured by static sensors at the time they were sampled.

## 1 Data Description

The water quality monitoring in Sitnica river in Kosova is performed through a Wireless Sensor Network (WSN) which supports remote, continuous and real-time measurements for the water quality parameters through its corresponding static sensors. Measured data (including the location and timestamp of the actual measurement) are in XML (Extensible Markup Language)) and CSV (Comma Separated Values) format transmitted via GPRS to the remote server at University of Prishtina, and stored dynamically in an MS SQL server. XML is an-easy to exchange format between different platforms and devices, as is CSV as a text format. Also for the sake of reusability, i.e. platform-independence, the dataset provided here is in text format (CSV).

The parameters measured remotely by the WSN static sensors are: temperature, electrical conductivity, pH, and dissolved oxygen. The WSN is installed in river bank Sitnica in village Plemetin located near Kosovo's capital city Prishtina, and measures water quality parameters in two points: sensing node 1 (housing) and sensing node 2 (manhole) in a distance of around 100 m from each other. The coordinates of each of the two sensing nodes are given in Table 1. The measurement period covered is almost eight months of continuous measurements in total starting May 2015 to beginning of January 2016. The frequency of measurements is configured to be real-time in intervals of every 10 min, given that configuration to a custom sampling time intervals is supported by the WSN installed. Sensors were at first calibrated.

**Table 1:** Coordinates of the sensing nodes 1 and 2 deployed in the Sitnica river bank.

Node Id	Longitude	Latitude
1	21.03843117	42.70670319
2	21.03802872	42.70727921

Table 2 shows an excerpt of the raw measurement data parameter-wise, i.e., one record for each parameter among temperature, pH, conductivity, and dissolved oxygen sensed by a given sensor node (column Node Id in the table) in a given time (column Timestamp). The corresponding file with the complete table, i.e. raw dataset parameter-wise, is available in [1] as a csv text and as an Excel table.

**Table 2:** An excerpt of raw measurement data.

Id	Node Id	Timestamp	Parameter	Value
1744	1	201505311809110000	pH	7.92
1745	1	201505311809110000	DissolvedOxygen	45.4
1746	1	201505311809110000	Conductivity	336.9
1747	1	201505311809110000	Temperature	16.43
80518	2	201505311858100000	pH	8.11
80519	2	201505311858100000	DissolvedOxygen	13.1
80520	2	201505311858100000	Conductivity	313.6
80521	2	201505311858100000	Temperature	16.61

There are in total 105652 measurements (records) registered with static WSN sensors. Basic statistics of raw measurement data are summarized in Table 3. Given there are 199 duplicates found among records in Node Id, Timestamp and Parameter columns, remaining are 105453 records. The Value column resulted duplicate as well among the identified duplicate records, which validates that for the given duplicate set of records, there is one same measurement value sensed for a given parameter by the same sensor node in the same time. Measurements conducted count to 78378 records or 74.33% in sensing node 1 (housing), and to 27075 records or 25.67% in sensing node 2 (manhole). The distribution among four distinct parameters of measurements performed is almost evenly as listed in the table.

**Table 3:** Basic statistics of raw measurement data.

#measurements	#measurements per node	#measurements per parameter
105453	Node 1 (housing): 78378	Conductivity: 26365
	Node 2 (manhole): 27075	DissolvedOxygen: 26364
		Temperature: 26364
		pH: 26360

Raw measurement data are further engineered to be appropriate in structure for analysis: grouped by Timestamp and Node Id (see SQL script in [1]). Hence, in the restructured dataset (Table 4), there is one common raw which provides values for all four parameters measured at the given time by a given sensor. Further, one new column Timestamp as DateTime has been introduced in the table, to show the Timestamp in the format easily recognized by the human reader. The corresponding file with the complete table, i.e. restructured dataset grouped by timestamp, is available in [1] as a csv text and as an Excel table.

**Table 4:** An excerpt of measurement data grouped by Timestamp and Node Id, and sorted by Node Id and Timestamp.

Id	Node Id	Timestamp	Timestamp as DateTime	Temperature	Conductivity	pH	DissolvedOxygen
17667	1	201512080246360000	2015/12/08 02:46:36 0000	6.29	274.8	6.11	92.3
17668	1	201512080256480000	2015/12/08 02:56:48 0000	6.13	265.7	6.11	93
17669	1	201512080306580000	2015/12/08 03:06:58 0000	6.24	276.2	6.12	92.4
17670	1	201512080317080000	2015/12/08 03:17:08 0000	6.29	275.8	6.13	91.5
25799	2	201506170132450000	2015/06/17 01:32:45 0000	20.08	392.1	6.94	39.2
25800	2	201506170142550000	2015/06/17 01:42:55 0000	20.04	390.8	6.71	39.8
25801	2	201506170153060000	2015/06/17 01:53:06 0000	20.03	392.1	6.96	38.8
25802	2	201506170203170000	2015/06/17 02:03:17 0000	20.05	393.3	6.96	40

There are in total 29842 records in the restructured dataset. Basic statistics of this dataset are summarized in Table 5. Outlier parameter values affect having notable misleading statistics in minimal, maximal and standard deviation for each of the parameters, but the remaining statistics in number of distinct values (column count), mean, and all three percentiles 25%, 50% and 75% per parameter are less affected and descriptive for the values of parameters available in the dataset.

**Table 5:** Basic statistics of measurement data.

	Id	Node Id	Timestamp	Temperature	Conductivity	pH	DissolvedOxygen
**count**	29842	29842	29842	26364	26365	26360	26364
**mean**	-	-	-	14.45	295.76	9.66	35.26
**std**	-	-	-	8.26	1867.65	16.94	36.17
**min**	-	-	-	-0.35	-145212.5	-28	0
**25%**	-	-	-	9.3	275	5.48	9.5
**50%**	-	-	-	15.6	324.7	6.31	19.3
**75%**	-	-	-	18.53	388.9	7.63	49.6
**Max**	-	-	-	115.42	26378.7	94.58	154.6

**Fig. 1:**
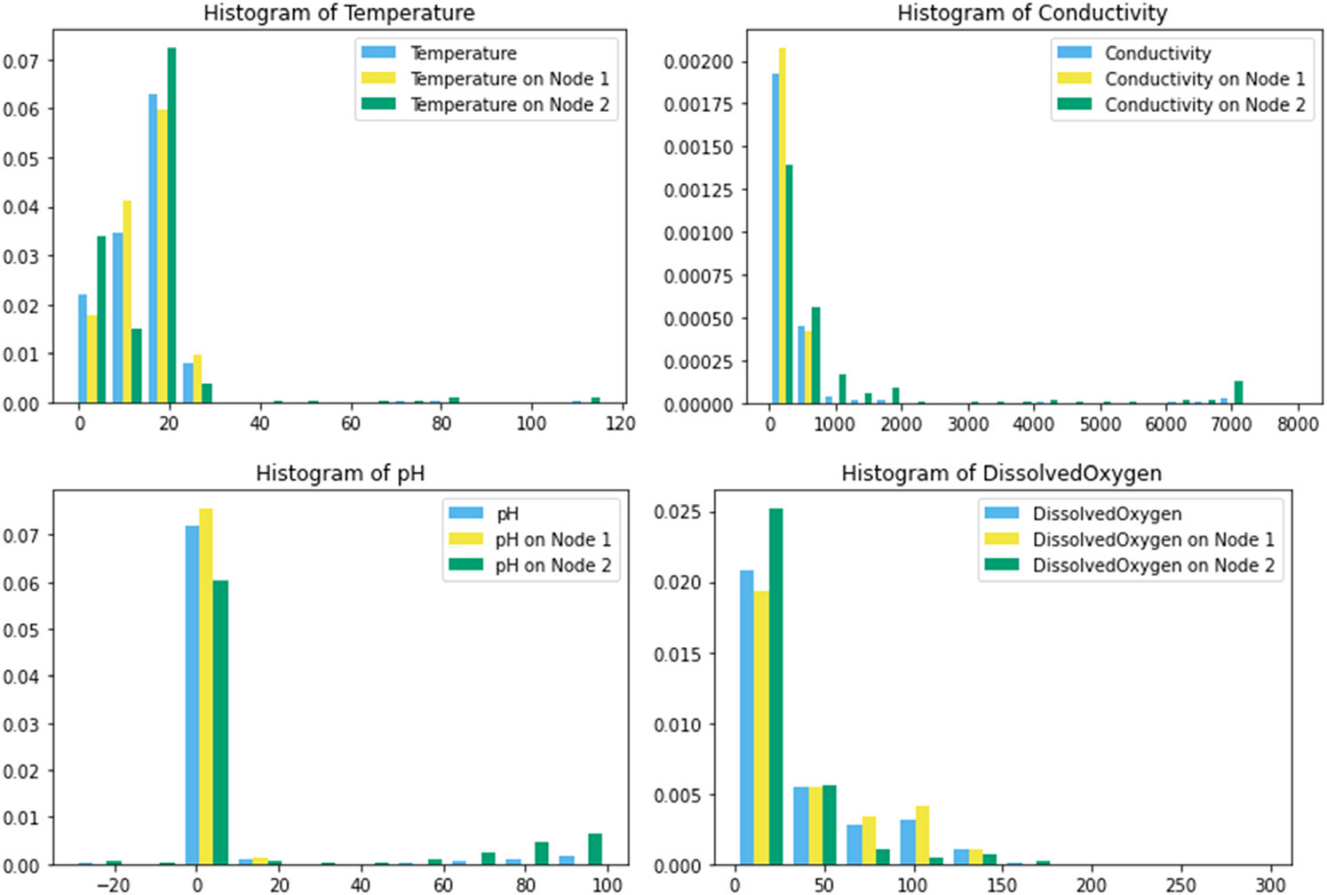
Histograms of individual water quality parameters.

Further, the distribution of values for each individual water quality parameter, including separately in both node 1 and node 2, is depicted in the histograms provided in Fig. 1.

## 2 Data Preprocessing

By merely observing the measurement data, few potential issues that were obvious to address in order for the dataset to be ready for use are data type, data consistency, missing data, and duplicate data.

*Task 1 (Data type problems).* The Timestamp column is of type integer, hence it is converted to datetime. The datatime format selected is '%Y%m%d%H%M%S%f', which means for instance that the value “201505011730590016” is converted to “2015-05-01 17:30:59.001600”.

*Task 2 (Data consistency problems).* Do we have consistent data? The Timestamp column, namely its values shall not range in the future. The Timestamp dates are confirmed to be consistent, i.e., no measurements occur “in the future”.

*Task 3 (Missing data problems).*  There are missing data in certain columns, and there is maybe a correlation in the missingness of the data.

**Fig. 2:**
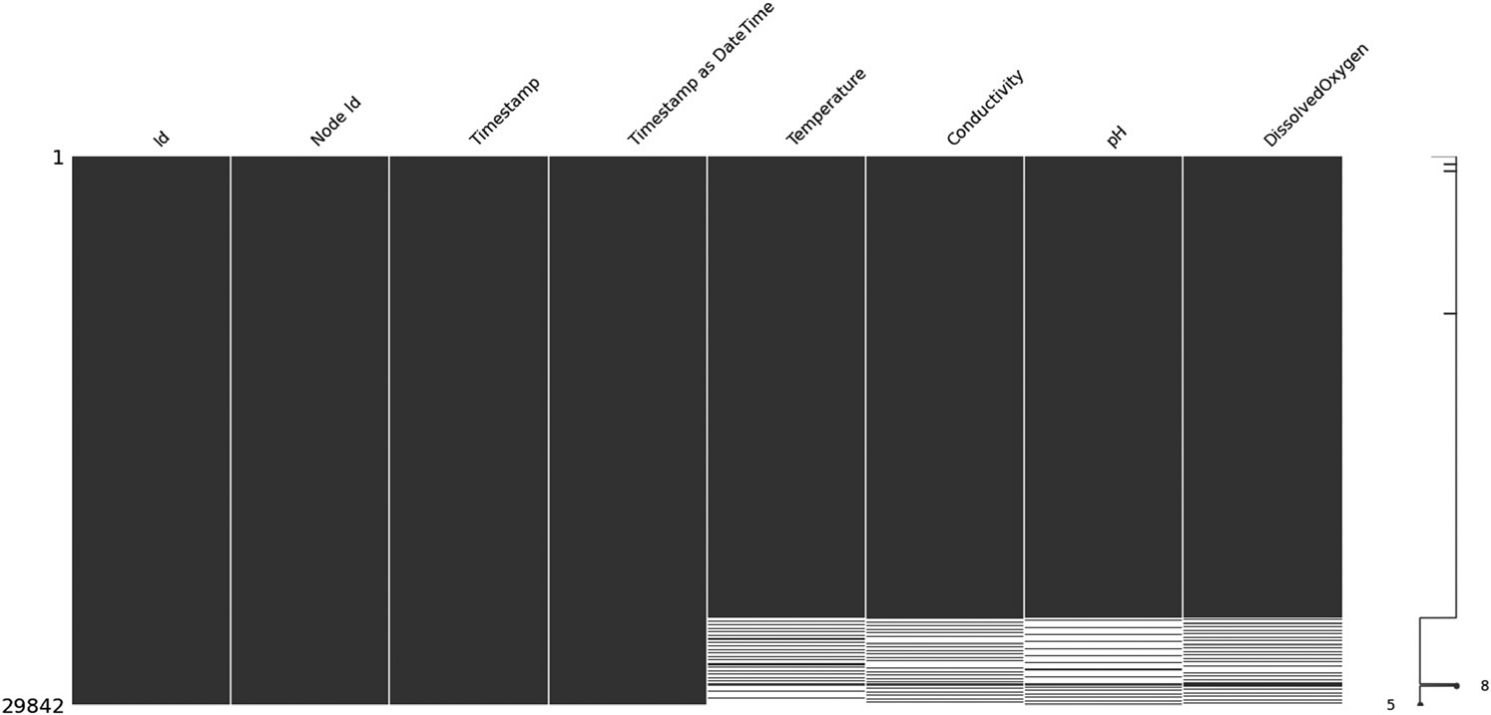
Missing values’ matrix sorted by Timestamp.

There are missing data in all four water quality parameters (Table 6), i.e., Temperature, Conductivity, pH, and DissolvedOxygen, and it seems that the missingness among these parameter values is related since they have almost same amount of missing data. Let's check for type of sensing node (node 1 or 2) and its relationship to missingness of parameter values. It is confirmed that *null* values appear only when node 1 measurements were performed. Moreover, it is evident from the missing values’ matrix depicted in Fig. 2 that null values appear sometimes by the end of the measurement period.

**Table 6:** Missing data across columns.

column name	#missing values
Node Id	0
Timestamp	0
Timestamp as DateTime	0
Temperature	3478
Conductivity	3477
pH	3482
DissolvedOxygen	3478

Next (Table 7) is some summary statistics on missingness of values in the dataset, where number of rows having null values in any of four parameters counts to 4642 rows, making some 18.42% of all rows in the dataset.

**Table 7:** Missing data summary statistics.

total #rows	all 4 parameters are not null	any of 4 parameters is null	all 4 parameters are null
29842	25200	4642	0

It is interesting to observe that *null* values could be resolved if timestamp data in the dataset rounded up to minutes, i.e., if ignoring the delays in seconds of emitting the measured parameter values from the sensors to the remote monitoring stations. As an illustration, in case of the following four rows (Table 8):

**Table 8:** An excerpt of measurement data with *null* values.

19165	1	201512222016418000	2015/12/22 20:16:41 8000	NULL	NULL	6.25	NULL
19166	1	201512222016431000	2015/12/22 20:16:43 1000	NULL	NULL	NULL	4.4
19167	1	201512222016440000	2015/12/22 20:16:44 0000	NULL	278.3	NULL	NULL
19168	1	201512222016481000	2015/12/22 20:16:48 1000	5.3	NULL	NULL	NULL

If the timestamp would be rounded to 2015/12/22 20:16 (Table 9):

**Table 9:** An excerpt of measurement data with *null* values and the Timestamp rounded to minutes.

19165	1	201512222016	2015/12/22 20:16	NULL	NULL	6.25	NULL
19166	1	201512222016	2015/12/22 20:16	NULL	NULL	NULL	4.4
19167	1	201512222016	2015/12/22 20:16	NULL	278.3	NULL	NULL
19168	1	201512222016	2015/12/22 20:16	5.3	NULL	NULL	NULL

than the above four rows would have been represented with one single row with no *null* values (Table 10):

**Table 10:** An excerpt of measurement data with no *null* values once the Timestamp rounded to minutes.

19165	1	201512222016	2015/12/22 20:16	5.3	278.3	6.25	4.4

These observations, namely null values evidence indicate that there might have been certain communication issues while transmitting data from the sensors in Plemetin village to the central remote monitoring node at University of Prishtina when measurements performed by a certain node (node 1) and at a certain time period (by the end of the measurement period, i.e. in winter).

*Task 4 (Duplicate data issues). Do we have duplicate data? Check if there are duplicate data in the dataset* and handle them properly. Check duplicates across all columns except the Id column which is unique for each row. There are no duplicate rows across all columns, given Id column is excluded.

### 2.1 Experimental Design, Materials, and Methods

This dataset was collected as part of the InWaterSense^1^, an R&D project supported by EU aimed to build a Wireless Sensor Network (WSN) in the river Sitnica for monitoring its water quality, and as a good practice to expand it to other surface water resources in the Republic of Kosovo in the future.

In the previous section, description of which parameters are measured, frequency in time of measurements, and coverage sensing area of measurements are already provided. The rationale behind why this river Sitnica and the selected site Plemetin are covered with WSN measurements is elaborated in the research article [2], as well as the system design and implementation. Next only an excerpt of the design and its implementation of the static WSN system is provided.

### 2.2 System Design and Implementation

Deployment of Wireless Sensor Networks for water quality monitoring is a pratice already apart from the traditional grab sampling approach [3], [4], [5], [6], [7]. The conceptual design of the WSN system deployed in Plemetin site for monitoring water quality in Sitnica, and consists of three types of nodes [2]:

• Static sensing nodes,
• A gateway node, and
• A central monitoring node.

There are two static sensing nodes in the WSN system in Sitnica river bank in Plemetin dislocated along two regions where measurements take place:

• On the discharge point source to which the discharge tube is extended, the measurements are performed with wireless static sensors placed at the wireless sensing node maintained from within the housing.
• Downstream the river in ca 100 m distance from the point source, the measurements are performed with wireless static sensors placed at the wireless sensing node maintained from within a manhole.

The gateway node in Plemetin site receives monitored data transmitted from static sensing devices via the ZigBee protocol and cables, and transmits them further via GPRS protocol remotely to the central remote monitoring node (i.e., server machine) in the premises of the University of Prishtina. Gateway devices are actually housed within the manhole of the WSN system in Plemetin. A database server machine installed in the laboratories at University of Prishtina, Faculty of Civil Engineering, Hydrotechnics Department, serves as a central monitoring node, and is connected to the gateway node in Plemetin site.

**Fig. 3:**
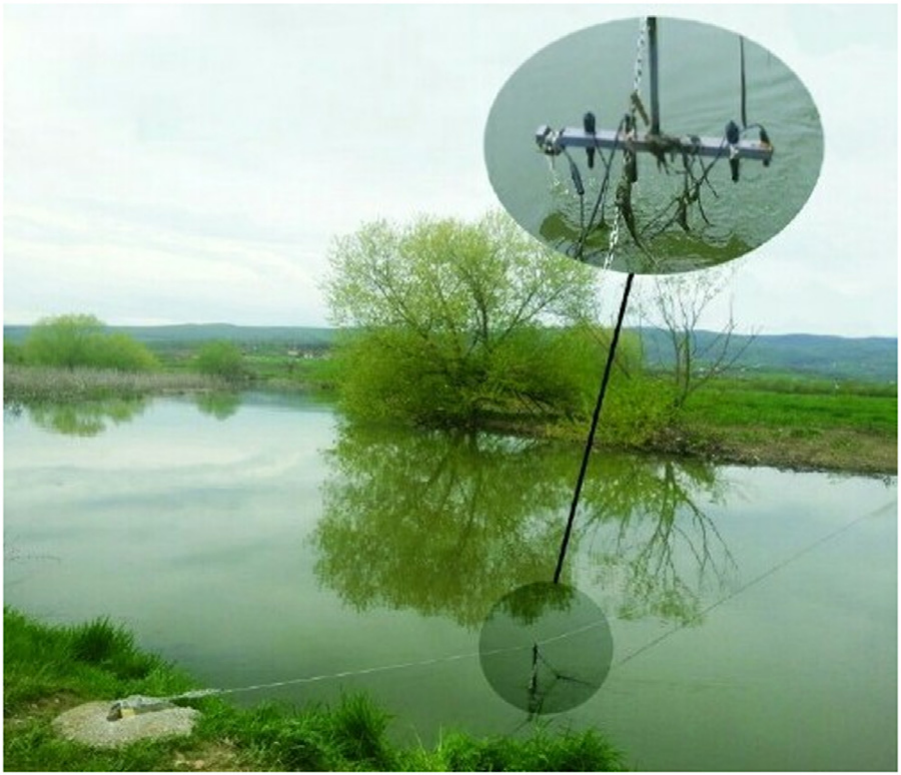
A static sensing node in Sitnica river comprised of wireless sensors (middle in the underwater). Adapted from [2].

Static sensing nodes comprise each of certain number of wireless sensors (Fig. 3) which measure water quality parameters as specified above, namely temperature, conductivity, pH, and dissolved oxygen.

## CRediT authorship contribution statement

**Figene Ahmedi:** Conceptualization, Methodology, Validation, Investigation, Resources, Data curation, Writing – original draft, Supervision, Project administration, Funding acquisition. **Lule Ahmedi:** Software, Formal analysis, Resources, Data curation, Writing – review & editing, Visualization, Project administration, Funding acquisition.

## Declaration of Competing Interest

The authors declare that they have no known competing financial interests or personal relationships which have, or could be perceived to have, influenced the work reported in this article.

## Data Availability

InWaterSense Dataset: Data from a wireless sensor network on water quality monitoring in a river in Kosovo (Original data) (Mendeley Data).

## Footnotes

1 http://inwatersense.uni-pr.edu/
